# Supplementary material for: Orally delivered MK-4482 inhibits SARS-CoV-2 replication in the Syrian hamster model
Source: Nat Commun. 2021 Apr 16;12:2295. doi: 10.1038/s41467-021-22580-8 (PMC8052374; doi:10.1038/s41467-021-22580-8)
Supplement: Supplementary file 1 — Supplementary Information [file 41467_2021_22580_MOESM1_ESM.pdf]

Supplementary Information: Orally delivered MK-4482 inhibits SARS-CoV-2 replication in the Syrian hamster model

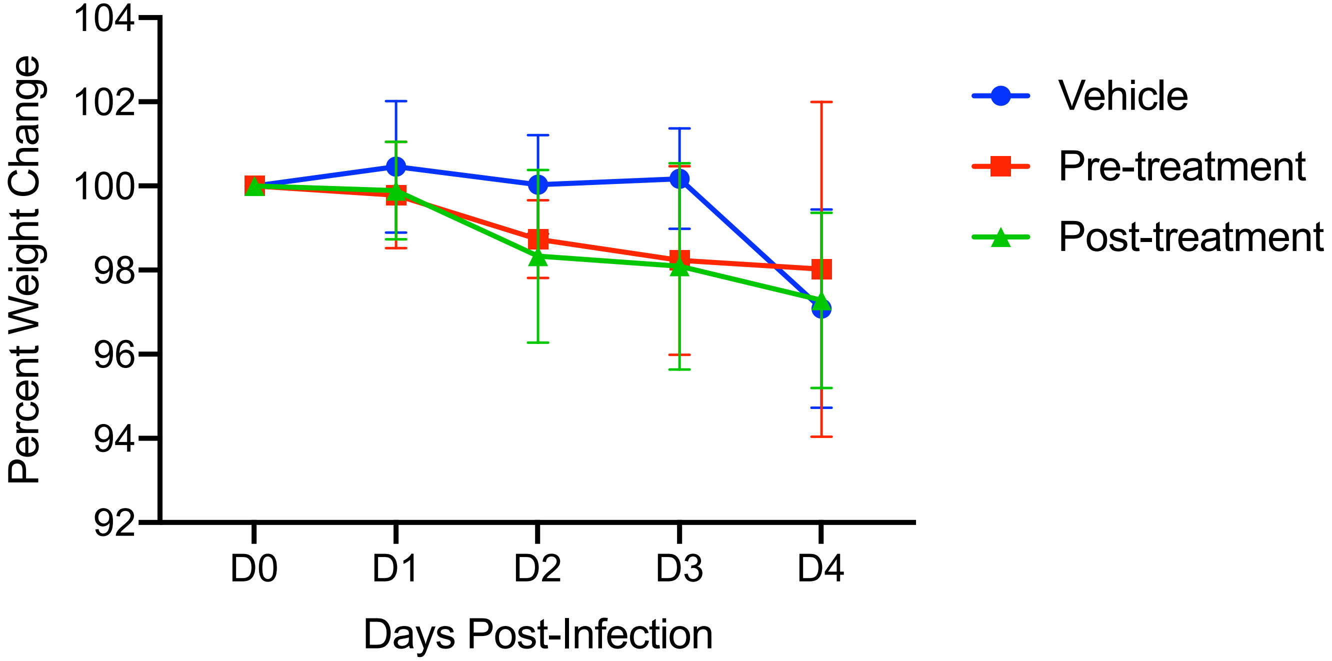

Supplementary Figure 1. Daily weight change of infected hamsters. Animals (N=6 per group) were weighed daily beginning on D0, pre-infection. Daily weights were compared to the initial weights to determine percent weight over the course of the study. Mean and SD are shown. One-way ANOVA followed by Kruskal-Wallis analysis and a pairwise Wilcox test was used to analyze differences among groups. There was no significant difference in weight loss between groups at any time.

	EIDD-1931 Lung Concentration						
	Vehicle control						Avg ± Std
nmol/gram	0	0	0	0	0	0	0
Estimated concentration µM	0	0	0	0	0	0	0
	Pre-treatment						Avg ± Std
nmol/gram	16.29	15.43	27.62	12.82	24.93	15.69	18.80 ± 5.97
Estimated concentration µM	13.03	12.34	22.1	10.25	19.95	12.55	15.04 ± 4.78
	Post-treatment						Avg + Std
nmol/gram	23.98	9.54	23.3	17.67	14.15	16.72	17.56 ± 5.49
Estimated concentration µM	19.18	7.63	18.64	14.14	11.32	13.37	14.05 ± 4.39

Supplementary Table 1. Lung concentrations of EIDD-1931. MK-4482 and the EIDD-1931 metabolite were measured in clarified lung homogenate by liquid chromatography and mass spectrometry (LCMS) at the point of necropsy. Concentration was estimated in lung based on the tissue being approximately 80% by weight to provide a conservative estimated EIDD-1931 lung concentration under the assumptions of homogenous distribution and hydration. Standard curves of MK-4482 and EIDD-1931 were made in lung homogenate from uninfected animals and subjected to irradiation to account for molecular degradation. All analytes were quantified against an 8-point calibration curve of the respective synthetic standard prepared in the target matrix and processed in the same manner as experimental samples. In line with its demonstrated rapid hydrolysis to EIDD-1931 following absorption, MK-4482 was not detected in the tissues. Limits of quantification in lung homogenate after irradiation was 5 ng/mL for EIDD-1931 and 50 pg/mL for MK-4482.

Substitution Rate of MK-4482

Substitution Type	I	Vehicle 1	Vehicle 2	Vehicle 3	Vehicle 4	Vehicle 5	Vehicle 6	Pre-treatment 1	Pre-treatment 2	Pre-treatment 3	Pre-treatment 4	Pre-treatment 5	Pre-treatment 6	Post-treatment 1	Post-treatment 2	Post-treatment 3	Post-treatment 4	Post-treatment 5	Post-treatment 6
A:T				1										1					
A:C				1															
A:G									2	7	17	3		3		1	1	1	2
T:A														1					
T:C										2	10	3	1	1		2		2	
T:G		1	1													1			
C:A															1				
C:T		1	3	3	1		1		4	13	4	9	8	2	2			2	
C:G																			
G:A									7	4	4	7	6		1	4		3	4
G:T										1									
G:C																			
Total	0	2	4	5	1	0	1	0	13	27	35	22	15	8	4	8	1	8	6

Substitutions per genome

0 5 10 15 20

Supplementary Table 2. Analysis of frequency and spectra of mutations in viral genome of MK-4482-treated and untreated animals. Viral RNA was isolated from lung samples of individual animals from different treatment groups (N=6 animals per group) followed by cDNA library preparation, sequencing and examination for mutations. Libraries were visualized on a BioAnalyzer DNA1000 chip and quantified on a CFX96 Real-Time System as detailed in Methods. Sequencing was performed on an Illumina NextSeq 550. Reads were paired up and aligned to the SARS-CoV-2 genome from isolate SARS-CoV-2/humanUSA/WA-CDC-WA1/2020 using Bowtie2 v 2.2.9. PCR duplicates were removed using Picard MarkDuplicates and variant detection was performed using GATK HaplotypeCaller with ploidy set to 2. Viral genome read depth coverage was greater than 100x for all samples. When compared to the vehicle, viral genomes from MK-4482 treated animals had a significant accumulation of nucleotide substitutions with an increased accumulation of adenosine-to-guanosine and cytosine-to-uracil transitions in viral genomes. Color ranges were selected with white representing 0 mutations and red representing 20 or more mutations. Colors intensity within the range was automatically calculated using the Prism (v8) software.

Molecule	MRM pair	DP (V)	EP (V)	CE (V)	CXP (V)
MK-4482	330.0/128.0*	70	10	20	15
MK-4482	330.0/110.0	60	10	40	15
EIDD-1931	260.0/128.0*	90	10	20	15
EIDD-1931	260.0/110.0	110	10	50	15

*Signal used for quantification

Supplementary Table 3. LCMS/MS MRM source conditions for the quantification of MK-4482 and EIDD-1931. MRM was performed using the indicated optimized conditions. To ensure signal fidelity triggered spectra were compared back to synthetic standards. Previously published MRM signals for biological nucleosides were utilized to confirm minimal interference at the retention time of interest as detailed in Methods.

Key: MRM: multiple reaction monitoring; DP: declustering potential; EP: entrance potential; CE: collision cell entrance potential; CXP: collision cell exit potential

	Type	Sequence: 5’ to 3’	Modifications
SARS-CoV-2_ NqPCR_Forward	Forward Primer	AGAATGGAGAACGCAGTGGG	None
SARS-CoV-2_ NqPCR_Reverse	Reverse Primer	TGAGAGCGGTGAACCAAGAC	None
SARS-CoV-2_ NqPCR_Probe	Probe	CGATCAAAACAACGTCGGCC	5’ 6-carboxyfluorescein Internal Zen quencher 3’ Iowa black quencher

Supplementary Table 4. List of primers used for quantitation of SARS-CoV-2. Real-time RT-PCR against the N gene using primers shown was used to quantify SARS-CoV-2 RNA as detailed in Methods.
