# Supplementary material for: Orally delivered MK-4482 inhibits SARS-CoV-2 replication in the Syrian hamster model
Source: Nat Commun. 2021 Apr 16;12:2295. doi: 10.1038/s41467-021-22580-8 (PMC8052374; doi:10.1038/s41467-021-22580-8)
Supplement: Supplementary file 3 — Reporting Summary [file 41467_2021_22580_MOESM3_ESM.pdf]

nature research

Corresponding author(s): Michael A Jarvis
Heinz Feldmann

Last updated by author(s): Feb 2, 2021

Reporting Summary

Nature Research wishes to improve the reproducibility of the work that we publish. This form provides structure for consistency and transparency in reporting. For further information on Nature Research policies, see our Editorial Policies and the Editorial Policy Checklist.

Statistics

For all statistical analyses, confirm that the following items are present in the figure legend, table legend, main text, or Methods section.

n/a	Confirmed	
☐	☒	The exact sample size (n) for each experimental group/condition, given as a discrete number and unit of measurement
☐	☒	A statement on whether measurements were taken from distinct samples or whether the same sample was measured repeatedly
☐	☒	The statistical test(s) used AND whether they are one- or two-sided<br>*Only common tests should be described solely by name; describe more complex techniques in the Methods section.*
☒	☐	A description of all covariates tested
☐	☒	A description of any assumptions or corrections, such as tests of normality and adjustment for multiple comparisons
☐	☒	A full description of the statistical parameters including central tendency (e.g. means) or other basic estimates (e.g. regression coefficient) AND variation (e.g. standard deviation) or associated estimates of uncertainty (e.g. confidence intervals)
☐	☒	For null hypothesis testing, the test statistic (e.g. F, t, r) with confidence intervals, effect sizes, degrees of freedom and P value noted<br>*Give P values as exact values whenever suitable.*
☒	☐	For Bayesian analysis, information on the choice of priors and Markov chain Monte Carlo settings
☒	☐	For hierarchical and complex designs, identification of the appropriate level for tests and full reporting of outcomes
☒	☐	Estimates of effect sizes (e.g. Cohen's d, Pearson's r), indicating how they were calculated

Our web collection on statistics for biologists contains articles on many of the points above.

Software and code

Policy information about availability of computer code

Data collection	Data was collected in Excel, v16.4 and then moved into Prism, v8. Statistical analysis was performed in R, v4.02. ImageScope Positive Pixel Count Algorith v9.1 was used for analysis of IHC lung sections. Cutadapt v1.12 combined with FASTX Toolkit was used for trimming and filtering raw fastq reads. Bowtie2 v2.2.9 combined with Picard MarkDuplicates v2.18.7 (Broad Institute) and GATK HaplootypeCaller v4.1.2.0 was used for further NGS analysis.
Data analysis	Significance was assessed by One-way ANOVA followed by a Kruskal-Wallis test and a pairwise Wilcoxon rank sum test to correct for multiple comparisons.

For manuscripts utilizing custom algorithms or software that are central to the research but not yet described in published literature, software must be made available to editors and reviewers. We strongly encourage code deposition in a community repository (e.g. GitHub). See the Nature Research guidelines for submitting code & software for further information.

Data

Policy information about availability of data

All manuscripts must include a data availability statement. This statement should provide the following information, where applicable:

- Accession codes, unique identifiers, or web links for publicly available datasets
- A list of figures that have associated raw data
- A description of any restrictions on data availability

SARS-CoV-2 isolate nCoV-WA1-2020 accession MN985325.1 (https://www.ncbi.nlm.nih.gov/nuccore/MN985325) was used for the study. The data that support the findings of this study are available from the corresponding author upon reasonable request. Source data are provided with this paper. Next generation sequencing data has been deposited in NCBI under BioProject accession number PRJNA691961 (https://www.ncbi.nlm.nih.gov/bioproject/PRJNA691961).

Field-specific reporting

Please select the one below that is the best fit for your research. If you are not sure, read the appropriate sections before making your selection.

- [x] Life sciences
- [ ] Behavioural & social sciences
- [ ] Ecological, evolutionary & environmental sciences

For a reference copy of the document with all sections, see nature.com/documents/nr-reporting-summary-flat.pdf

Life sciences study design

All studies must disclose on these points even when the disclosure is negative.

Sample size	Determination of animal sample size was based on the virological data from earlier studies. Groups of 6 hamsters are necessary to achieve statistical significance with a confidence interval of 95% for a two-fold difference in viral organ load. These studies were designed to assess the efficacy of the drug countermeasure utilizing the SARS-Cov-2 infection model. One group consisted of untreated animals to serve as an infection control. Two additional groups are used added to assess the timing of administration.
Data exclusions	No data was excluded
Replication	All attempts at duplication were successful. In brief, we used groups of 6 animals per treatment, with each animal serving as a replicate within the group. In addition, the use of multiple methods of analysis based on i) RT-PCR, i) infectious virus titration, and level of antigen positivity were used to independently confirm data from all animals.
Randomization	Male and female animals were randomly assigned groups, although each group had 3 males and 3 females.
Blinding	Down stream sample processing, pathology and data analysis was performed on coded samples. The sample code was not unlocked until the the samples were processed and data collected. The code was then unlocked to establish the final results.

Reporting for specific materials, systems and methods

We require information from authors about some types of materials, experimental systems and methods used in many studies. Here, indicate whether each material, system or method listed is relevant to your study. If you are not sure if a list item applies to your research, read the appropriate section before selecting a response.

Materials & experimental systems

n/a	Involved in the study
	x Antibodies
	x Eukaryotic cell lines
x	Palaeontology and archaeology
	x Animals and other organisms
x	Human research participants
x	Clinical data
x	Dual use research of concern

Methods

n/a	Involved in the study
x	ChIP-seq
x	Flow cytometry
x	MRI-based neuroimaging

Antibodies

Antibodies used	SARS-CoV/SARS-CoV-2 nucleocapsid antibody (Sino Biological cat#40143-MM05). The secondary antibody was the Vector Laboratories ImPress VR anti-mouse IgG polymer (cat# MP-7422).
Validation	Sino Biological cat#40143-MM05 has been validated by the manufacturer for use by WB and ELISA. Use of the antibody for detection of SARS-CoV-2 by IHC has been recently validated (Liu et al. JCI Insight 2020. 5: e139042). The secondary antibody has been used for detection of mouse IgG by IHC in multiple previous publications (see: https://vectorlabs.com/immpress-hrp-anti-mouse-igg-rat-adsorbed-peroxidase-polymer-detection-kit.html).

Eukaryotic cell lines

Policy information about cell lines

Cell line source(s)	Calu-3 and Vero-E6 cells were used in the study. Cells were originally obtained from the American Tissue Culture Cell (ATCC) repository.
Authentication	These cell lines were not authenticated.
Mycoplasma contamination	All cell culture stocks are checked and are mycoplasm free.

Commonly misidentified lines (See ICLAC register)	Search of the ICLAC Register of Misidentified Cell Lines (v.10) for Calu-3 and Vero-E6 did not identify any misidentified cell lines amongst the 552 registered lines that were of a concern.

Animals and other organisms

Policy information about studies involving animals; ARRIVE guidelines recommended for reporting animal research

Laboratory animals	Female and Male Syrian hamsters between 4-6 weeks old were used for the in vivo analysis.
Wild animals	No wild animals were used in the study
Field-collected samples	No field-collected samples were used in the study
Ethics oversight	Work with infectious SARS-CoV-2 complied with all relevant ethical regulations for animal testing and research. The hamster study received ethical approval from the Rocky Mountain Laboratories Animal Care and Use Committee (IACUC, Protocol # 2020-044-E) and was performed in a high biocontainment laboratory at Rocky Mountain Laboratories (RML), NIAID, NIH. Animal work was performed by certified staff in an Association for Assessment and Accreditation of Laboratory Animal Care International accredited facility. Work followed the institution's guidelines for animal use, the guidelines and basic principles in the NIH Guide for the Care and Use of Laboratory Animals, the Animal Welfare Act, United States Department of Agriculture and the United States Public Health Service Policy on Humane Care and Use of Laboratory Animals.

Note that full information on the approval of the study protocol must also be provided in the manuscript.
